# Editorial: Game changers in inflammatory bowel diseases, volume II

**DOI:** 10.3389/fmed.2023.1223473

**Published:** 2023-06-19

**Authors:** Barbara Dorottya Lovász, Anita Bálint

**Affiliations:** ^1^Institute of Applied Health Sciences, Semmelweis University, Budapest, Hungary; ^2^Department of Medicine and Oncology, Semmelweis University, Budapest, Hungary; ^3^Department of Medicine, University of Szeged, Szeged, Hungary

**Keywords:** IBD, EIM, tailored therapy, markers, systemic inflammation

The treatment of patients with inflammatory bowel diseases (IBDs) has undergone many changes recently. Novel therapeutic agents, medications, and endoscopic techniques have become available, however, the goal of long-term medical therapy remains the same—to achieve and maintain control over the inflammation and to induce remission against symptomatic or even asymptomatic disease activity.

The game changers in IBD nowadays center around how can we reach these goals in everyday clinical practice and how we can succeed in creating an effective tailored therapy for patients.

A combination of medical therapies can be an option for patients who suffer from the most severe and complicated phenotypes of IBD. Combinative or concomitant use of biological therapies can fill gaps in these cases, but it is not an instant solution for everyone. Many factors can influence this efficacy bidirectionally, e.g., the nutritional state, body composition, and body mass index. It is important to know the disease course, the risk factors of progression, and the development of complications to achieve an optimal and effective therapy for IBD patients.

Shehab et al. investigated this question in a cross-sectional study, analyzing how biological therapies impact the body mass index of IBD patients. This study was performed in an IBD tertiary care center, and it searched for associations between BMI, type of IBD, and the type of medications taken among patients with IBD of varying weight categories. Patients received tumor necrosis factor antagonist combination and anti-integrin therapies. The primary outcome was the association between BMI and medication used in IBD. Many patients with IBD are either obese or overweight. Patients with IBD who are on anti-TNF combination therapy or vedolizumab monotherapy are more likely to be obese and overweight. In addition, patients with ileocolonic CD are more likely to be obese or overweight. These pieces of information may be important in the selection of biological therapies.

The next interesting way forward in tailored therapy is how we can combine the therapies and what kind of biochemical markers or activity indexes can help to predict the disease course of IBD or even the disease activity before the appearance of clinical symptoms.

Pan et al. investigated the role of seven peripheral marker ratios in blood in the diagnosis and the prediction of disease activity. Their data suggest the neutrophil-to-prealbumin ratio (NPAR) as a putative biomarker for diagnosing and predicting disease activity in patients with IBD.

Song et al. analyzed the interleukin-26 (IL-26) expression in IBD and its immunoregulatory effects on macrophages. Compared with healthy controls, in IBD patients, IL-26 levels were elevated in the inflamed intestinal mucosa and reduced in the peripheral blood. The transcriptional changes in macrophages exposed to IL-26 suggest that IL-26 may amplify the aberrant immune response in IBD by activating macrophages.

The disease course can be extremely different from patient to patient, and some of the patients can be at higher risk of special comorbidities, e.g., systemic inflammatory conditions.

A systematic review and meta-analysis written by Domokos et al. investigated the specific relationship between two multifactorial diseases: inflammatory bowel disease and periodontitis. Patients with periodontitis were more susceptible to IBD, and periodontitis was significantly associated with the risk of subsequent UC but not with subsequent CD. So patients with IBD have a higher chance of developing periodontitis and are a high-risk population in dentistry. Multidisciplinary team management is very important in the complex therapy of patients with IBD. The authors emphasize the importance of prevention and that both dentists and gastroenterologists should be aware of this relationship.

Migration is also an important question in the wider aspects of patient management regarding the change of the disease phenotype and course. In a prospective, observational, multicenter study by Gutiérrez et al., the ENEIDA-registry was used to compare cohorts of IBD patients who were born outside Spain with a cohort of native patients. The main ethnic groups of immigrant IBD patients in Spain are Caucasian, Latin American, and Arab. Immigrant IBD patients in Spain are younger and have more extraintestinal manifestations than native-born patients. Accordingly, the use of biologics is more frequent among immigrants. Compared with native-born patients, first-generation-immigrant IBD patients in Spain were younger at disease onset and showed an increased risk of having extraintestinal manifestations and using biologics. This study suggests a featured phenotype of immigrant IBD patients in Spain and constitutes a new landmark in the epidemiological characterization of immigrant IBD populations in southern Europe.

Patients with IBD can be at higher risk for liver injuries due to the side effects of medications, even the use of over-the-counter (OTC) drugs, or due to extraintestinal manifestations. In a meta-analysis and systematic review by Wang et al., the authors aimed to investigate the risk of liver injury in patients with IBD and those with non-IBD diseases treated with methotrexate (MTX). Patients with IBD receiving methotrexate are at higher risk of liver injury compared with patients with non-IBD diseases. The authors concluded that IBD is associated with a higher risk of MTX-related liver injury. The mechanism of MTX-induced hepatotoxicity might be different in IBD and non-IBD diseases and needs to be verified in future research.

The editors hope that the readers of the present volume of Game Changers in IBD have found some novel interesting results in the land of study of the mysterious IBD to reach the optimal tailored therapy for patients.

## Author contributions

All authors listed have made a substantial, direct, and intellectual contribution to the work and approved it for publication.

